# Delayed percutaneous coronary intervention for an extensive iatrogenic left main coronary artery dissection: a case report

**DOI:** 10.1186/s13256-022-03677-0

**Published:** 2022-12-05

**Authors:** Charalampos Varlamos, Efthymia Varytimiadi, Despoina-Rafailia Benetou, Dimitrios Alexopoulos

**Affiliations:** 2nd Department of Cardiology, Attikon University Hospital, National and Kapodistrian University of Athens Medical School, Rimini 1, Chaidari, 12462 Athens, Greece

**Keywords:** Left main coronary artery dissection, Iatrogenic dissection, Coronary angiography, Percutaneous coronary intervention, Case report

## Abstract

**Background:**

Iatrogenic left main coronary artery dissection is a rare but serious complication that can occur both during diagnostic coronary angiography and percutaneous coronary intervention. Early diagnosis and choice of optimal management are of crucial importance for patient’s outcome while representing a challenge for clinicians.

**Case presentation:**

We present a case of iatrogenic left main coronary artery dissection occurring during diagnostic coronary angiography in a 53-year-old Greek woman with a history of coronary artery bypass grafting. Although dissection was greatly extending to mid left anterior descending artery, delayed percutaneous coronary intervention was successfully performed by carefully wiring the true lumen.

**Conclusions:**

Delayed percutaneous coronary intervention, performed 25 days following the index event, proved to be a feasible and effective strategy for treating a widely extended left main coronary artery iatrogenic dissection.

**Supplementary Information:**

The online version contains supplementary material available at https://doi.org/10.1186/s13256-022-03677-0.

## Background

Iatrogenic dissection of left main coronary artery (LMCA) is a potentially life-threatening complication of coronary catheterization procedures, with incidence less than 0.1% [1]. It can occur during either diagnostic coronary arteriography (CA) or percutaneous coronary intervention (PCI), while its prompt identification and treatment continues to represent a challenge for interventional cardiologists [2].

## Case presentation

**Fig. 1:**
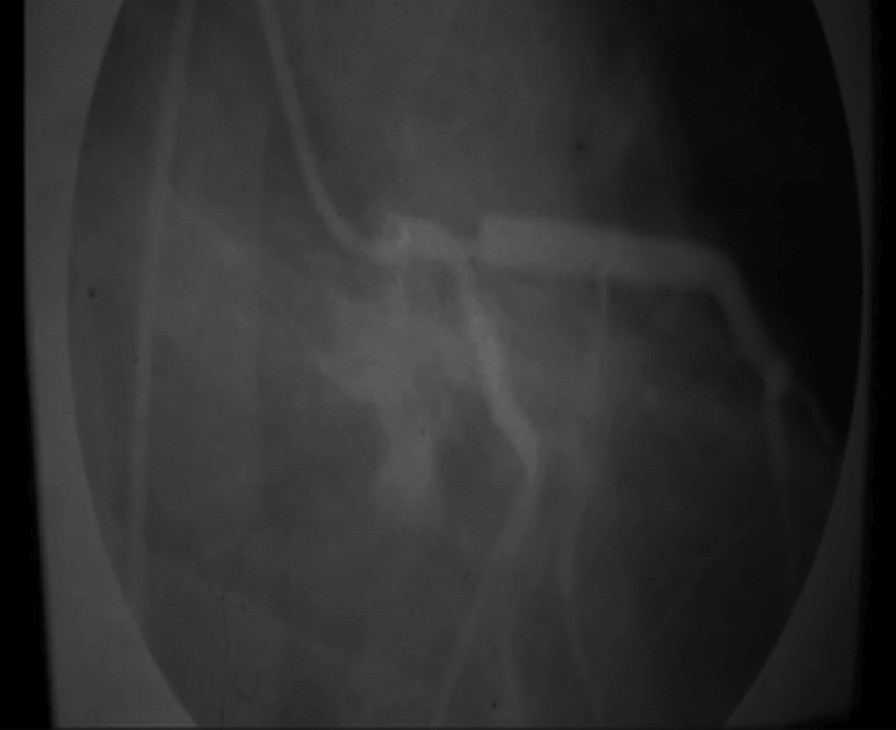
Frame from old coronary angiography showing the culprit bifurcation lesion leading our patient to coronary artery bypass grafting (CABG)

A 53-year-old Greek woman, with a history of coronary artery bypass grafting (CABG), presented to a local hospital complaining of angina at rest. At initial evaluation, electrocardiographic abnormalities were absent and troponin levels were within normal limits. Regarding her past medical history, patient suffered from a myocardial infarction 21 years ago. Her CA at the time was indicative of LMCA bifurcation disease (Fig. 1), and thus she was subsequently treated with CABG [left internal mammary artery (LIMA) to left anterior descending (LAD), right internal mammary artery (RIMA) to second obtuse marginal branch]. She also had a history of breast cancer treated with surgery, chemotherapy, and radiotherapy 8 years ago, active smoking, and dyslipidemia. She was occasionally on carvedilol and aspirin.

**Fig. 2:**
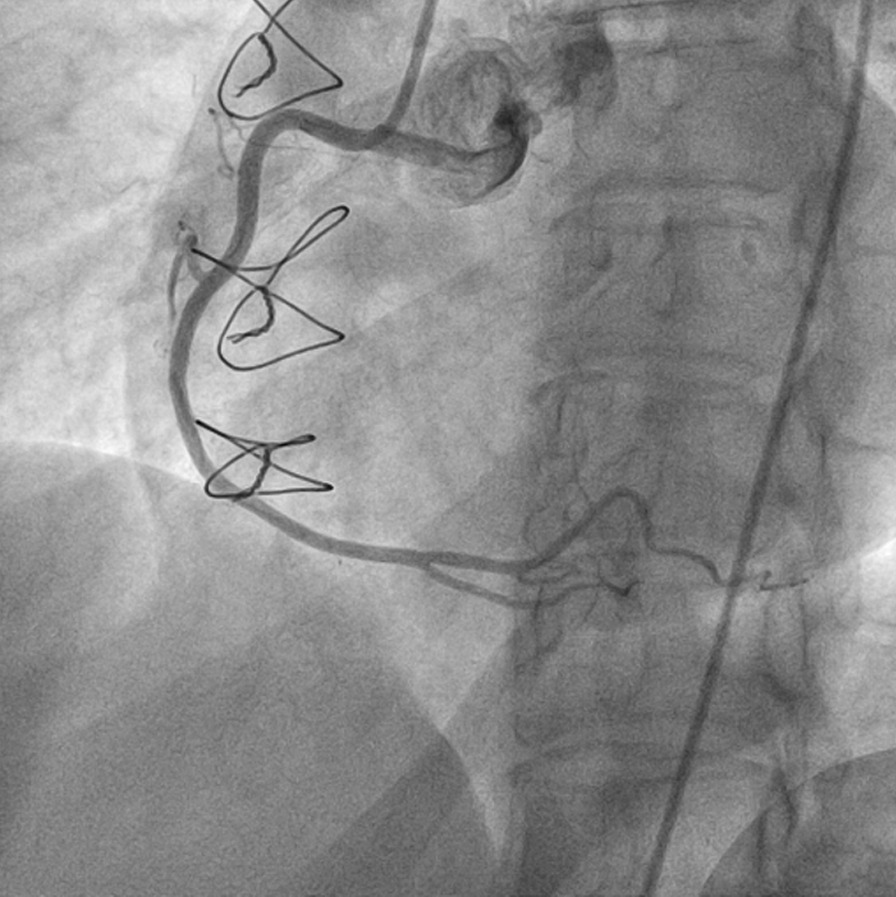
Right coronary artery with no significant lesions

**Fig. 3:**
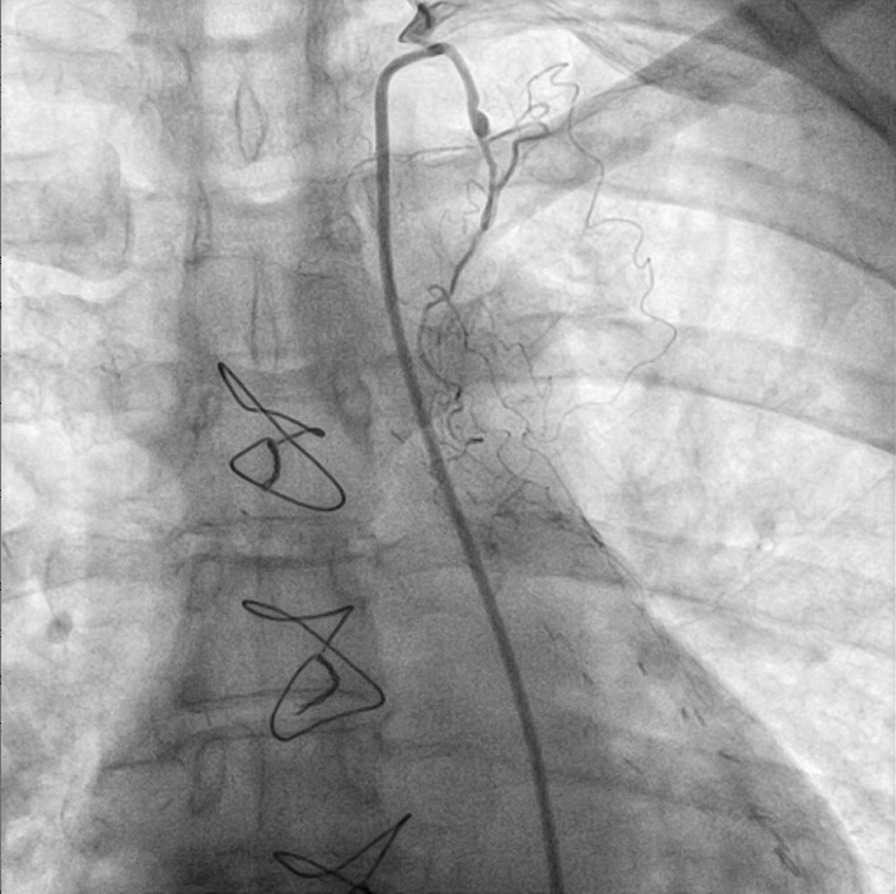
Degenerated, nonfunctional, used left internal mammary artery (LIMA)

**Fig. 4:**
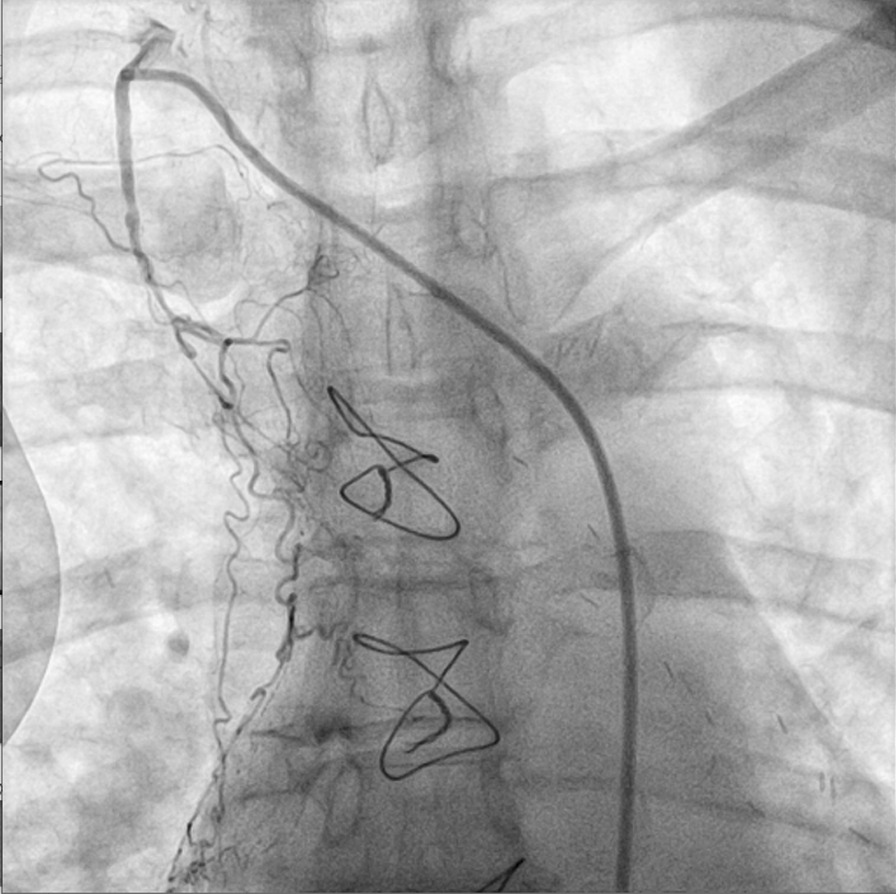
Degenerated, nonfunctional, used right internal mammary artery (RIMA)

On the basis of her worrisome symptoms and aforementioned medical history, patient was transferred to a university hospital with availability of a catheterization laboratory. Transthoracic echocardiogram did not reveal any regional wall motion abnormalities. A diagnostic coronary angiography (CA) revealed no significant stenoses in native coronary arteries (Additional file 1: Video 1, Additional file 2: Video 2, Fig. 2) as well as nonfunctional, degenerated left internal mammary artery (LIMA) and right internal mammary artery (RIMA) grafts (Figs. 3 and 4). During the procedure, patient complained of chest pain, accompanied by hemodynamic instability. Repeat evaluation of the left coronary system revealed a type B dissection of left main coronary artery (LMCA) (Additional file 3: Video 3). An effort for bail-out percutaneous coronary intervention (PCI) was unsuccessful. Thus, an intraaortic balloon pump was inserted and patient was transferred to the intensive care unit (ICU) for further management. Transthoracic echocardiogram revealed apical akinesis, electrocardiogram showed dynamic T wave changes and peak troponin levels rose to 530 pg/ml. Chest pain was sustained for a couple of hours but patient was soon stabilized and remained mildly symptomatic (with chest discomfort and mild dyspnea) during her ICU stay until she was eventually weaned off the pump and vasopressors.

**Fig. 5:**
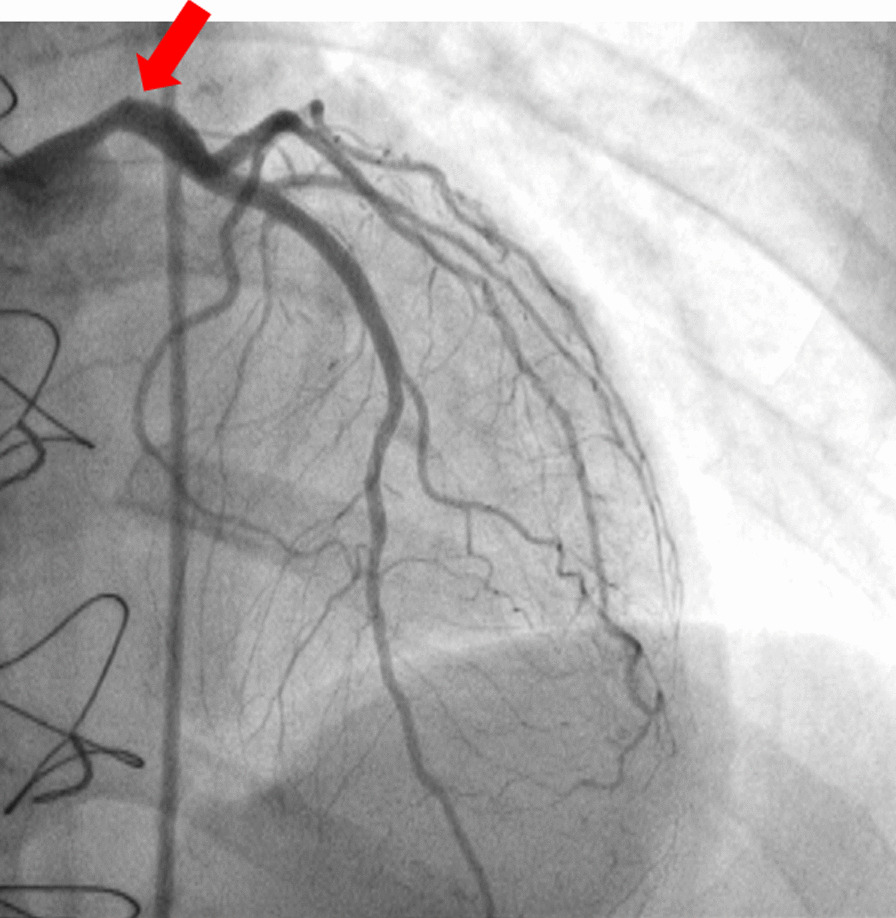
Deep intubation of Judkins left diagnostic catheter and contrast injection against the arterial wall (arrow)

**Fig. 6:**
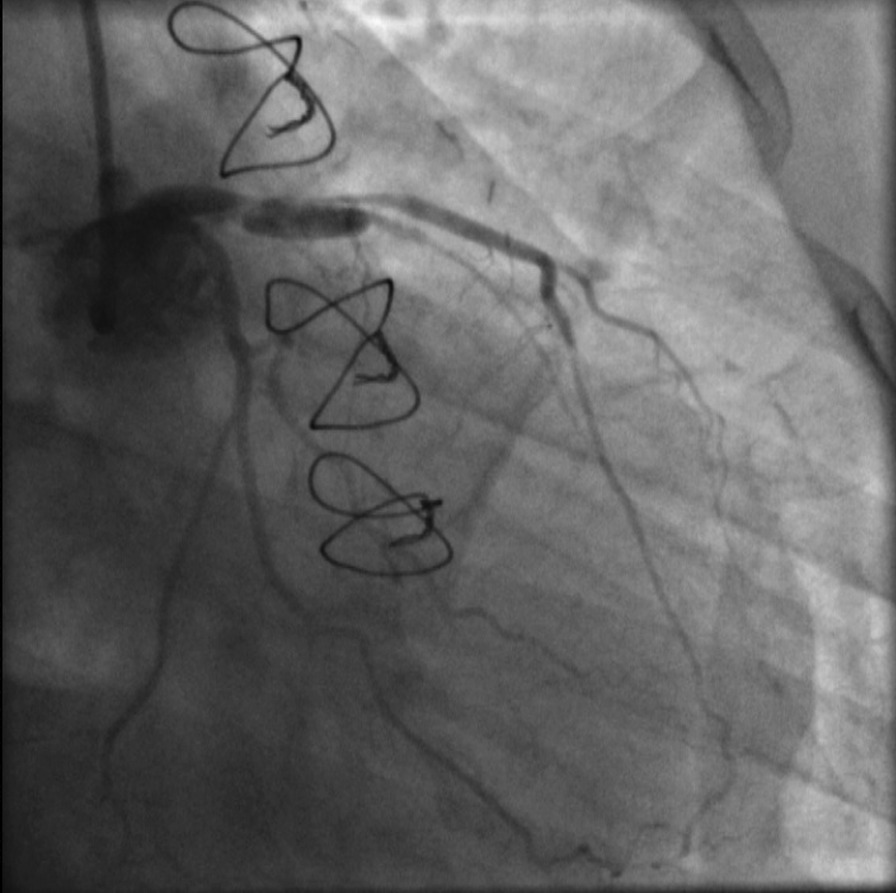
Reassessment of the left main coronary artery (LMCA) dissection 25 days after the initial event, revealing a worsened, extended lesion with a TIMI II flow in left anterior descending (LAD) artery

**Fig. 7:**
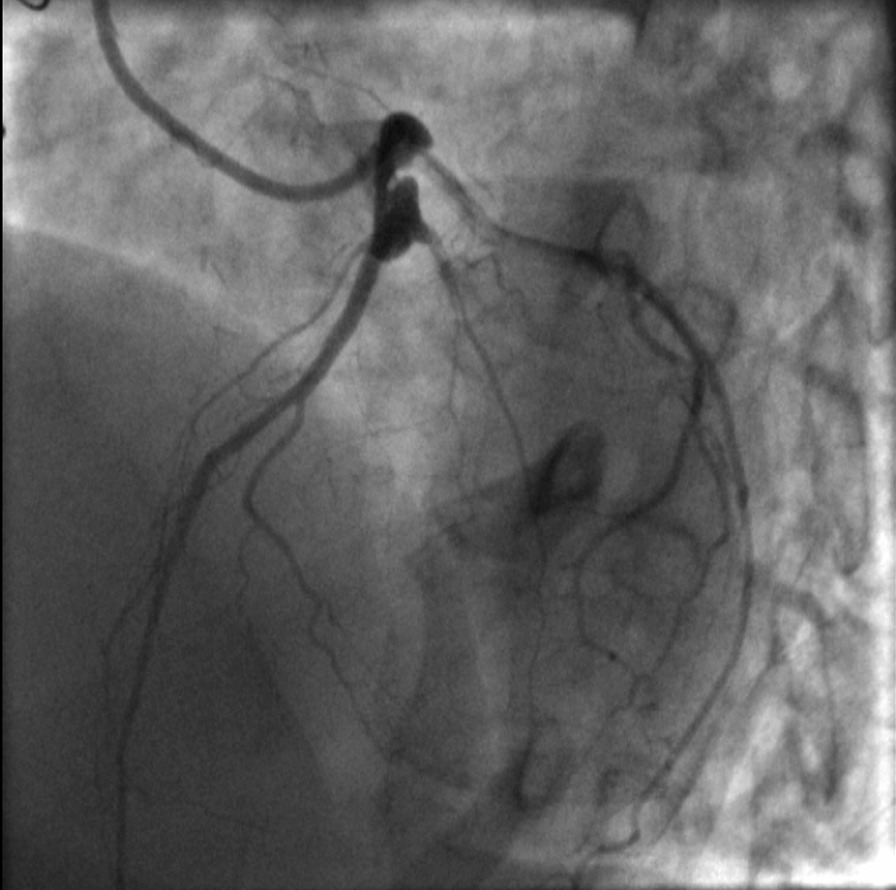
Another angiographic view indicative of the extensive LMCA dissection

Patient was discharged 25 days later and admitted to our department for further management. At presentation, she was mildly symptomatic; blood pressure was 117/63 mmHg, and heart rate was 89 beats per minute. Brief physical and neurological examination did not reveal any pathologic findings. Electrocardiographic evaluation revealed poor progression of R-wave and T-wave inversion in precordial leads; high sensitivity troponin levels were mildly elevated [32 pg/ml (upper normal limit 13 pg/ml)]. Renal and hepatic function were normal. Left ventricular ejection fraction (LVEF), assessed via echocardiography, was 50% with apical akinesis. Careful evaluation of her previous CA revealed deep “sitting” of the Judkins Left (JL) diagnostic catheter, with its tip pointing against the upper LMCA arterial wall, a probable cause of iatrogenic dissection (Fig. 5). A new CA confirmed the previously diagnosed LMCA dissection but of greater extent, from ostial LMCA to mid left anterior descending artery (LAD). True lumen of LAD was severely compressed by the false lumen, resulting in a thrombolysis in myocardial infarction (TIMI) II flow distally (additional file 4: video 4, additional file 5: video 5, additional file 6: video 6 and Figs. 6, 7).

**Fig. 8:**
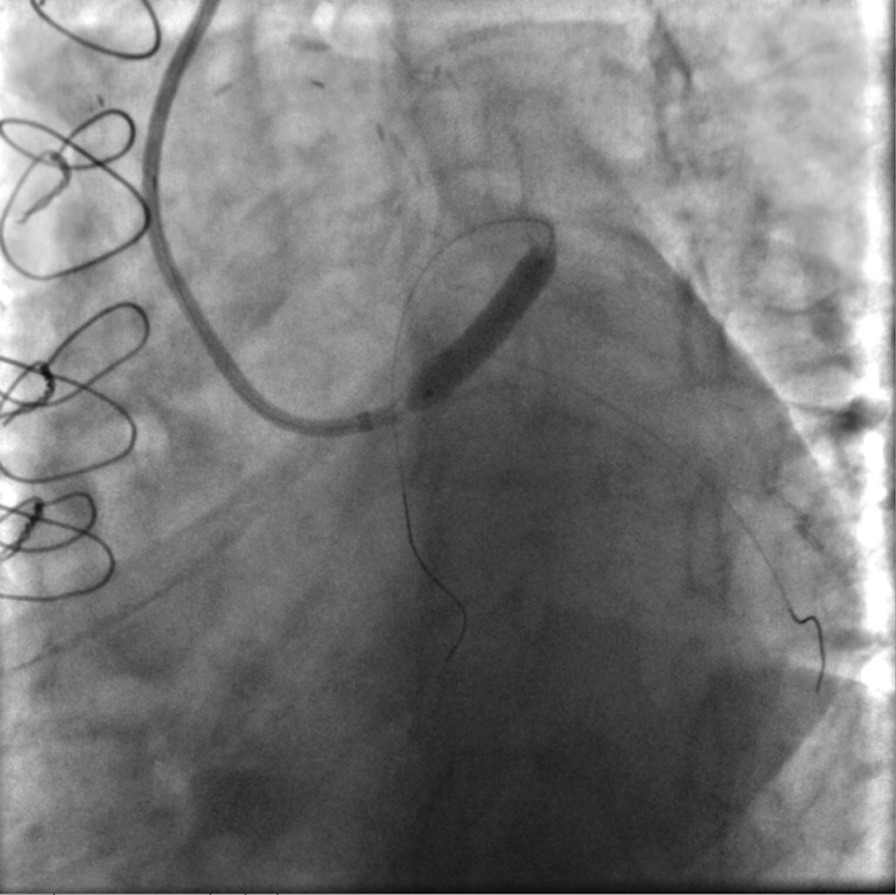
Stent deployment

**Fig. 9:**
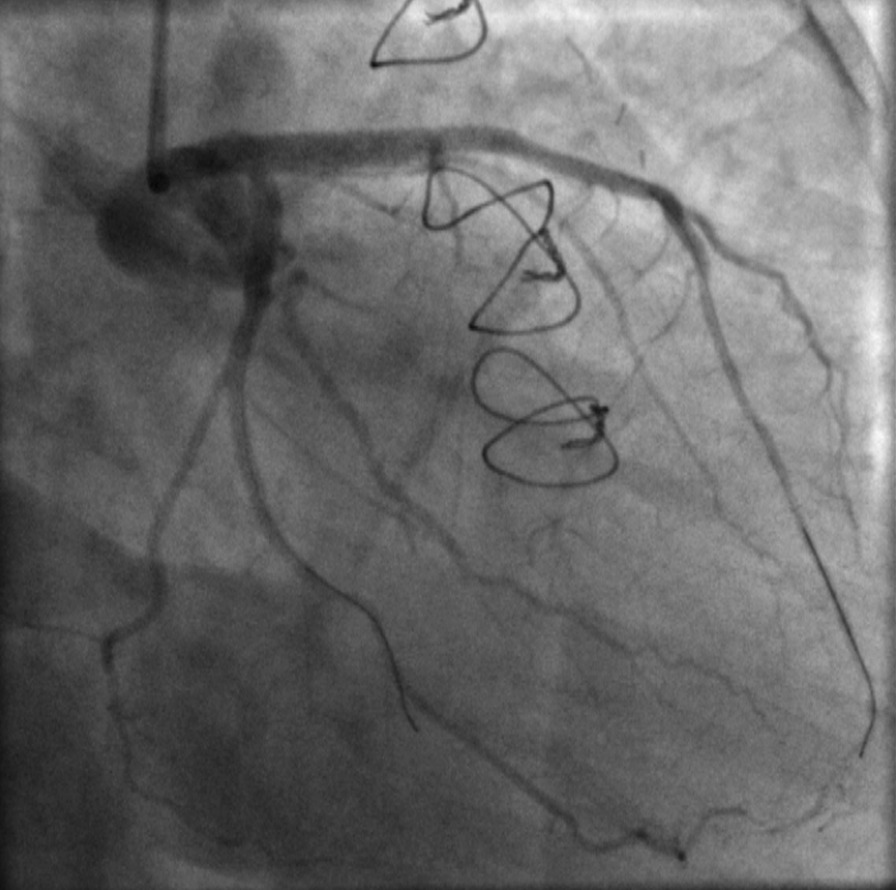
The final angiographic result

We decided to perform PCI to LMCA and LAD. A 6 Fr JL3.5 Launcher (Medtronic Inc., Minneapolis, MN, USA) guiding catheter was advanced to LMCA and a 0.014 in Asahi Sion blue support guidewire (Boston Scientific, Natick, MA, USA) was passed through the true lumen of LAD (additional file 7: video 7). Following predilatation with a 3 × 20 mm NC Solarice balloon (Medtronic Inc., Minneapolis, MN, USA), a 3 × 24 mm Synergy stent (Boston Scientific) was implanted to LAD and a 4 × 26 mm Resolute Integrity RX (Medtronic Inc., Minneapolis, MN, USA) stent was implanted proximally to the LMCA (Fig. 8). Post-dilatation up to 20 atmospheres was performed with a 3.5 × 12 mm NC Solarice balloon (Medtronic Inc., Minneapolis, MN, USA). The final angiographic evaluation revealed improved flow in LAD without compromising the ostium of left circumflex artery (additional file 8: video 8, additional file 9: video 9, Fig. 9). Patient had an uneventful recovery and was discharged the following day on ticagrelor 90 mg twice daily, aspirin 100 mg once daily, carvedilol 12.5 mg twice daily, rosuvastatin 40 mg once daily, and ramipril 2.5 mg once daily.

At follow-up visit at 6 months, patient was asymptomatic and in good functional status, with an LVEF of 50% assessed by echocardiography.

## Discussion

We herein describe a case-report of a 53-year-old woman, treated with PCI to LMCA and LAD for iatrogenic dissection, 25 days following the event. Of interest, patient reported a history of CABG for LMCA disease 21 years ago, but such a lesion was not evident in repeat angiographic evaluation.

In general, iatrogenic LMCA dissection is a serious complication, associated with underlying atherosclerotic disease, female sex, and complex PCI [3]. However, as in our patient’s case, it can also be provoked by suboptimal catheterization techniques, such as deep catheter intubation into LMCA and vigorous manipulations, as well as contrast injections toward the arterial wall [1, 4]. JL3.5 diagnostic catheter, which was used in our patient’s angiography, is the second most frequently associated with dissection catheter after EBU3.5 guide [5]. Owing to JL3.5 catheter’s shape, its tip was “scraping” against the upper arterial wall of our patient’s LMCA, not coaxially engaging the ostium, leading to a type B iatrogenic dissection [6].

Regarding management, we decided to proceed with PCI on the basis of patient’s previous surgical history and already used, though nonfunctional, IMA grafts, prior chest radiotherapy, and extent of disease. Conservative management as well as CABG represent alternative strategies, though PCI is the most commonly preferred technique for treating iatrogenic dissection [5, 7]. Although the dissection had occurred 25 days before, PCI was deemed to be the optimal treatment strategy on the basis of worse angiographic findings; further deterioration would lead to devastating effects for our patient since LMCA was affected, resulting in flow disturbance of LAD. Nevertheless, technical challenges of PCI include the identification and guidewire crossing of true lumen with the risk of extending the dissection. Of note, a brief management algorithm for catheter-induced iatrogenic dissection was recently released, including intravascular imaging for treatment guidance as well as bail-out stenting strategies [8]. Despite the lack of evidence in delayed revascularization of iatrogenic dissection, in our case, PCI to LMCA proved to be a feasible and effective treatment option, as also shown in other clinical scenarios [9].

Of interest, the culprit lesion of LMCA bifurcation (Fig. 1), leading to CABG more than two decades ago, was not present in our patient’s first diagnostic CA. Both grafts were nonfunctional, a finding that could be attributed to the fact that native coronary arteries had no significant stenoses and the antegrade flow was normal. On the basis of the above, the significant lumen narrowing that led our patient to surgery could be indicative of coronary artery spasm, dissection, or thrombus, rather than stable atherosclerotic plaque [10].

Our case highlights the importance of evaluating the nature of left main coronary lesions, especially in cases of younger women or patients without prominent coronary atherosclerotic disease. Prior to decision-making regarding treatment approach, nitrates should be administered to differentiate spasm from other causes.

## Conclusions

Early recognition of iatrogenic LMCA dissection and choice of appropriate treatment strategy is crucial, according to the type of dissection, the hemodynamic status of the patient, and the operator’s skills. Our patient, treated with CABG and with no apparent obstructive coronary artery disease in subsequent angiographic evaluation, suffered from an iatrogenic LMCA dissection during diagnostic CA that put her life at risk. Redo CABG was not an ideal option, and bail-out PCI was performed 25 days later even though the dissection had greatly extended.

## Learning objectives

- Iatrogenic left main coronary artery dissection is a life-threatening complication.
- It can be provoked during either diagnostic coronary angiography or percutaneous coronary intervention.
- Bail-out PCI is a feasible and effective solution in expert centers.
- Various disorders, apart from atherosclerotic disease, can lead to left main lumen narrowing and compromised coronary blood flow, especially in young women.

## Supplementary Information

**Additional file 1, video 1:** Evaluation of our patient’s left coronary system (RAO caudal view). Interestingly, left main bifurcation stenosis is not apparent.

**Additional file 2, video 2:** Second angiographic view of our patient (RAO cranial view). The deep intubation of the JL3.5 diagnostic catheter and injection of contrast against the wall is noted.

**Additional file 3, video 3:** A type B iatrogenic dissection of left main coronary artery (LMCA) is observed (RAO cranial view).

**Additional file 4, video 4:** Reevaluation of patient’s left coronary system (RAO caudal view) in our cath lab 25 days later, revealing a worsened, extended dissection from LMCA to mid LAD, with a TIMI II flow.

**Additional file 5, video 5:** Another view of the dissection (LAO cranial view), showing its extension from ostial left main to mid left anterior descending artery.

**Additional file 6, video 6:** Identification of double lumen, true and false, of the vessel (LAO caudal view).

**Additional file 7, video 7:** Wiring of true lumen (RAO caudal view).

**Additional file 8, video 8:** Final result (LAO caudal view).

**Additional file 9, video 9:** Final result (RAO caudal view). Of note, the flow in left circumflex artery was not compromised by the intervention in left main coronary artery (LMCA).

## Data Availability

Not applicable.

## Abbreviations

CA: Coronary angiography
CABG: Coronary artery bypass grafting
ICU: Intensive care unit
JL: Judkins left
LAD: Left anterior descending
LIMA: Left internal mammary artery
LMCA: Left main coronary artery
LVEF: Left ventricular ejection fraction
NHLBI: National Heart, Lung and Blood Institute
PCI: Percutaneous coronary intervention
RIMA: Right internal mammary artery
TIMI: Thrombolysis in myocardial infarction
